# Adaptive Human *CDKAL1* Variants Underlie Hormonal Response Variations at the Enteroinsular Axis

**DOI:** 10.1371/journal.pone.0105410

**Published:** 2014-09-15

**Authors:** Chia Lin Chang, James J. Cai, Shang Yu Huang, Po Jen Cheng, Ho Yen Chueh, Sheau Yu Teddy Hsu

**Affiliations:** 1 Department of Obstetrics and Gynecology, Chang Gung Memorial Hospital Linkou Medical Center, Chang Gung University, Kweishan, Taoyuan, Taiwan; 2 Department of Veterinary Integrative Biosciences, Texas A&M University, College Station, Texas, United States of America; 3 Reproductive Biology and Stem Cell Research Program, Department of Obstetrics and Gynecology, Stanford University School of Medicine, Stanford, California, United States of America; University of Bremen, Germany

## Abstract

Recent analyses have identified positively selected loci that explain differences in immune responses, body forms, and adaptations to extreme climates, but variants that describe adaptations in energy-balance regulation remain underexplored. To identify variants that confer adaptations in energy-balance regulation, we explored the evolutionary history and functional associations of candidate variants in 207 genes. We screened single nucleotide polymorphisms in genes that had been associated with energy-balance regulation for unusual genetic patterns in human populations, followed by studying associations among selected variants and serum levels of GIP, insulin, and C-peptide in pregnant women after an oral glucose tolerance test. Our analysis indicated that 5′ variants in *CDKAL1, CYB5R4*, *GAD2*, and *PPARG* are marked with statistically significant signals of gene–environment interactions. Importantly, studies of serum hormone levels showed that variants in *CDKAL1* are associated with glucose-induced GIP and insulin responses (*p*<0.05). On the other hand, a *GAD2* variant exhibited a significant association with glucose-induced C-peptide response. In addition, simulation analysis indicated that a type 2 diabetes risk variant in *CDKAL1* (rs7754840) was selected in East Asians ∼6,900 years ago. Taken together, these data indicated that variants in *CDKAL1* and *GAD2* were targets of prior environmental selection. Because the selection of the *CDKAL1* variant overlapped with the selection of a cluster of *GIP* variants in the same population ∼11,800 to 2,000 years ago, we speculate that these regulatory genes at the human enteroinsular axis could be highly responsive to environmental selection in recent human history.

## Introduction

Because of the recency of our common ancestry, it is well accepted that the investigation of positively selected single nucleotide polymorphisms (SNPs) in human populations may explain arrays of phenotypic variation in humans. Indeed, recent studies have identified hundreds of loci that exhibit evidences of positive selection in human genomes, and uncovered variants that describe variations in appearance, physiological adaptations, and pathological responses to diseases, thereby providing a better understanding of the molecular mechanisms that underlie these selections [1], [2], [3], [4]. However, genetic variants that confer adaptations in energy-balance regulation remain underexplored. Because such variants were likely subject to selection pressures that fluctuated over time, a moderate signal of selection could be all that has remained as a clue to selection. Accordingly, we screened gene region SNPs for unusual patterns in derived allele frequency and linkage disequilibrium (LD) in genes that had been associated with energy-balance regulation (i.e., past clinical investigations, genome-wide association (GWA) studies, and quantitative trait loci analyses) [5], [6].

In these studies, we identified common 5′ variants in five genes—glucose-dependent insulinotropic polypeptide (*GIP*), Cdk5 regulatory associated protein 1-like 1 (*CDKAL1*), cytochrome b5 reductase 4 (*CYB5R4*), glutamate decarboxylase 2 (*GAD2*), and peroxisome proliferator-activated receptor gamma (*PPARG*)—as top metabolic modifier candidates. With the exception of *GAD2* variant rs2236418, none of the candidate variants had been implicated in earlier GWA studies although *CDKAL1, CYB5R4*, and *PPARG* loci have been associated with diabetes- and/or obesity-related traits [7], [8], [9], [10], [11], [12]. It is possible that these variants operate under physiological conditions that have not been specifically investigated. Consistent with the hypothesis, our recent studies have shown that three regulatory *GIP* variants were positively selected ∼8,100 (11,800 to 2,000) years ago, and are associated with variations in glucose metabolism and glucose-induced GIP response in pregnant women; moreover, a coding *GIP* variant (rs2291725) affects the bioactivity of GIP [5], [6].

To gain a better understanding of whether and how *CDKAL1, CYB5R4*, *GAD2*, and *PPARG* variants contribute to adaptations in energy-balance regulation, we further explored their evolutionary history and potential genotype–phenotype relationships in humans.

## Materials and Methods

### Ethics Statement and Patients

The study was approved by the institutional ethics committee review board of Chang Gung Memorial Hospital Linkou Medical Center, and was conducted in accordance with the guidelines in The Declaration of Helsinki. A total of 131 unrelated healthy women with normal pregnancy were recruited, and all patients gave written informed consent to participate in the study. Related patients (i.e., blood relatives) were excluded to ensure that the observed associations were not due to confounding effects from ancestry. The screening glucose challenge test for gestational diabetes mellitus (GDM) was performed as previously described [13]. Specifically, patients were given a 50-gram glucose solution after overnight fasting at 23–29 weeks of gestation, and a blood sample was taken at 1 hour after glucose intake. The mean age and body mass index (BMI) of patients were 30.7±0.4 years and 25.4±0.3, respectively.

### Genotyping

Genomic DNA samples of subjects were extracted and purified from anticoagulated blood with the DNeasy Blood & Tissue Kit (Qiagen). Genotyping of SNPs was performed using the Applied Biosystems TaqMan Validated SNP Genotyping Assays. The genotyping analysis had a >96% success rate and >99% reproducibility.

### Measurements and statistical analysis of circulatory levels of GIP, insulin, and C-peptide

Blood samples were collected from patients 1 hr after administration of the oral glucose tolerance test (OGTT). Serum GIP, insulin, and C-peptide levels were measured using specific ELISA kits from Millipore, Mercodia, and Calbiotech, respectively. Patients' serum hormone profiles were analyzed by the χ^2^ test, the Student's *t*-test, or linear regression analysis using GraphPad Prism 5. All *p*-values were two-sided. The statistical significance cutoff value was 0.05.

### F_ST_ estimation and analysis of haplotypes

To identify adaptive SNPs, we screened for common gene region variants that have divergent allele frequencies in HapMap populations in 207 candidate genes [14]. To improve the odds of identifying potential genotype–phenotype relationships, we focused on SNPs with a >30% minor allele frequency (MAF) in the overall Eurasian (CEU [U.S. residents with northern and western European ancestry] and ASN [pooled samples of Chinese from Beijing [CHB] and Japanese from Tokyo [JPT]) populations. The population genetic differentiation statistic F_ST_ among HapMap II populations (i.e., CEU, ASN, and YRI [Yoruba from Ibadan]) was computed using the PGEToolbox and SPSmart [15], [16]. Putative positive SNPs (i.e., those in the top 10%) were further analyzed using pairwise population comparisons. The use of F_ST_ is advantageous given that F_ST_ measures the proportion of total genetic variance that is caused by differences among populations, and does not require assumptions about the structure of human populations and SNP ascertainment bias. In addition, LD blocks and haplotype plots were analyzed using HaploView 4.1 [17].

### Analysis of EHH and iHS statistics

We computed the extended haplotype homozygosity (EHH) statistic for SNPs as previously described [18], and the EHH plots were generated as described [4]. The EHH curve depicts the decay of identity of haplotypes that carry alleles of a core SNP as a function of distance between tested SNPs and the core SNP. When an allele rises rapidly in frequency due to positive selection, it tends to exhibit a high haplotype homozygosity that extends further than expected in a neutral model. For the analysis of EHH decay, haplotypes in the genomic region centered on select SNPs were downloaded from the HapMap project website.

The integrated Haplotype Score (iHS) statistic detects whether the area under the EHH curve for a selected allele is greater than that for a neutral allele; it is a measure of recent positive selection for variants that have not yet reached fixation [4]. To test whether the observed iHS deviated significantly from the expected neutral values, we used a coalescent model and generated 10,000 replicated haplotype sets using the coalescent simulator ms [19]. We adopted simulation parameters compatible with the sample size of the population and the length of the genomic region that was analyzed. The simulation was conditioned based on the same recombination rate and the number of segregating sites within tested regions.

### Estimation of the age of selected allele

When a novel allele is positively selected, the allele and its linked haplotype quickly rise to a high frequency. As the allele ages, the length of the haplotype shortens over time due to recombination and mutation. By modelling this process and measuring the decay of the haplotype that carries the positively selected allele, the age of the allele can be estimated [20]. The breakdown of the intactness of the haplotype surrounding the selected allele was modeled using a Poisson process as described earlier [21]. We obtained the recombination map from the FTP site of the HapMap project (ftp://ftp.ncbi.nlm.nih.gov/hapmap/recombination/). The rs7754840 block consists of 70 SNPs, and is represented by 11 distinct haplotypes in ASN; among which haplotypes 1, 6, and 8–10 carry the derived allele C of rs7754840 (Fig. S1 in File S1, upper left panel). Among these derived haplotypes, haplotype 1 with a frequency of 0.259 is apparently the ancestral haplotype, from which haplotypes 6 and 8–10—with frequencies of 0.029, 0.018, 0.015, and 0.015, respectively—were derived. The relative portion of haplotype 1 (the ancestral haplotype) of all derived haplotypes is *P*' = 0.77.

The recombination map correlates the increment of physical distance (Mb) with that of genetic distance (cM), which is derived from the estimates of LD between HapMap SNPs [22]. We took the mutation rate of the region *u* = 1.66×10^−6^—based on the haploid mutation rate of 1.1×10^−8^ per base per generation [23] and the probability of ascertaining HapMap SNPs, ∼10^−3^—for the analysis. We assumed that the most common haplotype is the ancestral haplotype and used *P*' as an approximation of *P*.

The probability that a haplotype remains ancestral (i.e., that the haplotype that carries the derived allele retains the status of high frequency right after being selected) is 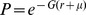, where *G* is the number of generations, *r* is the recombination rate per generation, and *μ* is the mutation rate per generation. For a given allele in the derived haplotype, the haplotype-decay approach estimates the number of generations *G* in terms of *P* (the probability that a given haplotype does not change from its ancestor) [20], [21].

In these analyses, the hypothetical demographic history assumed that the ASN population is one panmictic population that underwent a size reduction, followed by a period of constant size. The population had an ancestral population size of N1, which at time T2 instantaneously shrank to size N2. It remained constant at size N2 until time T1, at which point it began expanding exponentially until the present time. The population size at the present time is N3 (i.e., the bottleneck model) [24].

## Results

### Common 5′ variants in *CDKAL1*, *CYB5R4*, *GAD2*, and *PPARG* exhibit signals of selection

Studies of allele frequency of genic region SNPs in 207 candidate genes showed that, in addition to a cluster of 37 linked *GIP* SNPs [6], common variants in the 5′ proximate region of *CDKAL1* (rs9368197, position -1305 nt), *CYB5R4* (rs1325471, position -1695 nt), *GAD2* (rs2236418, position -243nt), and *PPARG* (rs2920502, position −154 nt) exhibit highly skewed population frequency in YRI, CEU, and ASN populations (Table 1) [5], [14]. The differences in derived allele frequency between African (YRI) and Eurasian (i.e., CEU and ASN) populations are in excess of 45–70%. For example, the derived allele of *PPARG* rs2920502 increased from 2.6% in YRI to 35.7% and 75.3% in CEU and ASN, respectively.

**Table 1:** Gene region candidate SNPs that exhibited signs of selection.

Gene (Candidate SNPs)	Association with disease risk^a^	Posi-tion (hg18)	Frequencies of derived alleles			Frequency differentiation (F_ST_)	Allele (anc/der)	Extended haplotype homozygosity
			YRI	CEU	ASN			
***CDKAL1*** (rs9368197)	T2D, ulcerative colitis, GDM, Crohn's, OB	Chr. 6, 20641k	0.051	0.233	0.511	F_ST (YRI-CEU)_ = 0.11 F_ST (YRI-ASN)_ = 0.44 F_ST (CEU-ASN)_ = 0.14	G/T	iHS _(YRI)_ = N.D. iHS _(CEU)_ = −0.67^c^ iHS _(ASN)_ = −1.24^c^
***CYB5R4*** (rs1325471)	Near a T2D susceptibility locus on Chr. 6	Chr. 6, 84624k	0.143	0.958	0.715	F_ST (YRI-CEU)_ = 0.77^b^ F_ST (YRI-ASN)_ = 0.45 F_ST (CEU-ASN)_ = 0.19	A/G	iHS _(YRI)_ = 1.41^c^ iHS _(CEU)_ = 0.43 iHS _(ASN)_ = −0.41
***GAD2*** (rs2236418)	OB, alcohol dependence, schizophrenia	Chr. 10, 26545k	0.126	0.836	0.704	F_ST (YRI-CEU)_ = 0.67^b^ F_ST (YRI-ASN)_ = 0.51 F_ST (CEU-ASN)_ = 0.04	G/A	iHS _(YRI)_ = −1.16^c^ iHS _(CEU)_ = 0.92^c^ iHS _(ASN)_ = 0.48
***PPARG*** (rs2920502)	T2D, obesity, coronary artery diseases	Chr. 3, 12304k	0.026	0.357	0.753	F_ST (YRI-CEU)_ = 0.28 F_ST (YRI-ASN)_ = 0.76^b^ F_ST (CEU-ASN)_ = 0.27	C/G	iHS _(YRI)_ = N.D. iHS _(CEU)_ = 0.52 iHS _(ASN)_ = 0.61
**Known type 2 diabetes- and GDM-associated intronic variants**								
***CDKAL1*** (rs7754840)	T2D, ulcerative colitis, GDM, Crohn's, OB	Chr. 6, 20769k	0.667	0.336	0.405	F_ST (YRI-CEU)_ = 0.18 F_ST (YRI-ASN)_ = 0.10 F_ST (CEU-ASN)_ = 0.02	G/C	iHS _(YRI)_ = 0.44 iHS _(CEU)_ = 0.04 iHS _(ASN)_ = −0.90^c^
***CDKAL1*** (rs7756992)	T2D, ulcerative colitis, GDM, Crohn's, OB	Chr6, 20787k	0.418	0.721	0.496	F_ST (YRI-CEU)_ = 0.23 F_ST (YRI-ASN)_ = 0.03 F_ST (CEU-ASN)_ = 0.10	G/A	iHS _(YRI)_ = 0.53 iHS _(CEU)_ = 0.13 iHS _(ASN)_ = 0.50

a T2D  =  Type 2 diabetes; GDM  =  Gestational diabetes mellitus; OB  =  obesity.

b Significant F_ST_, which is defined as higher than 95^th^ percentile of F_ST_ of common SNPs in the HapMap populations.

c Significant iHS assessed using coalescent simulations (see Methods). iHS has not been determined (N.D.) for SNPs with a minor allele frequency <0.10.

**Figure 1:**
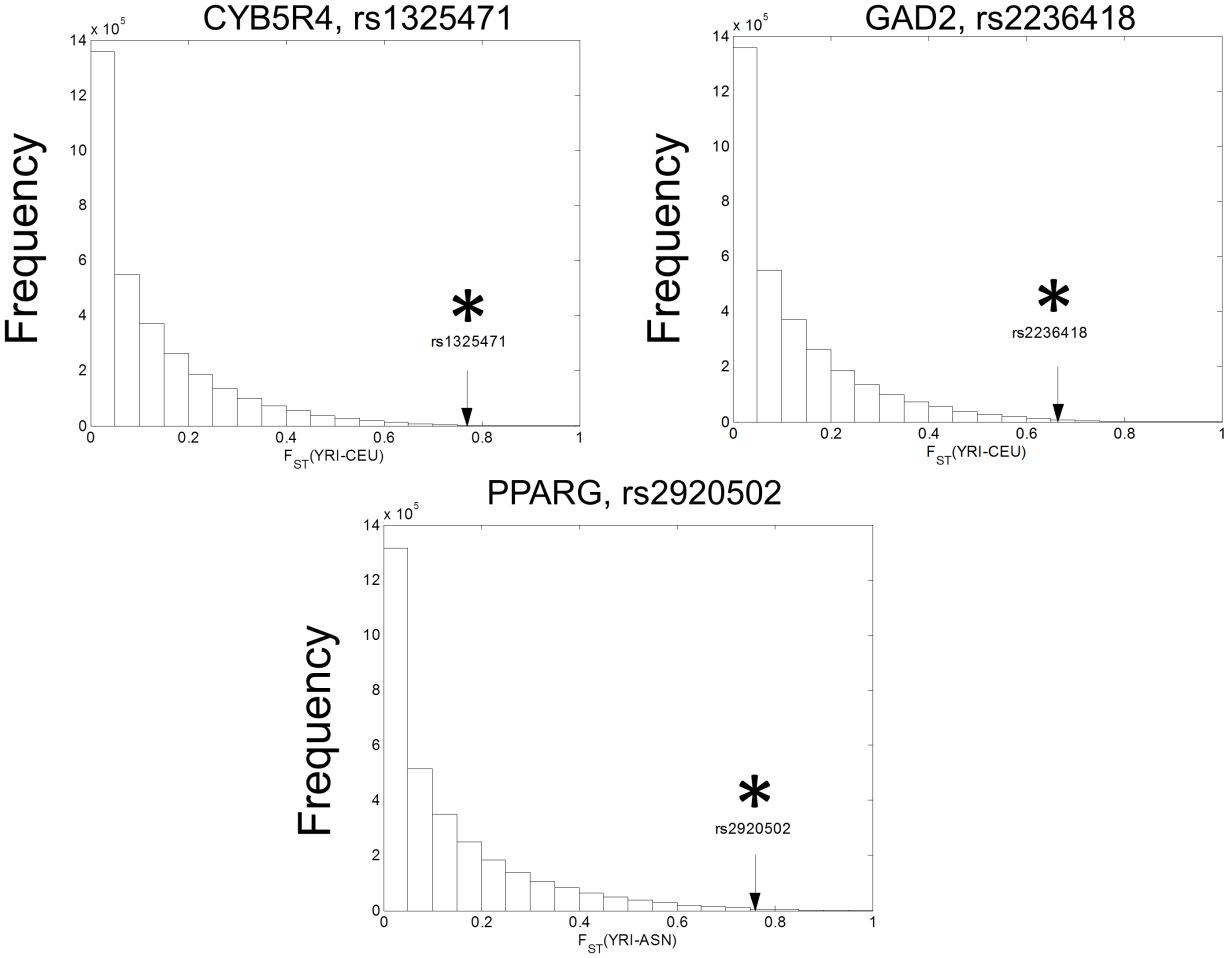
Differential distribution of *CYB5R4, GAD2*, and *PPARG* variants in human populations. The distribution of the F_ST_ for common SNPs across the human genome and the F_ST_ for rs1325471, rs2236418, and rs2920502. For rs1325471 and rs2236418, F_ST_ estimations between YRI and CEU (**a-b**) are shown on the *x*-axis. For rs2920502, comparisons between YRI and ASN (**c**) are shown on the *x*-axis. The black arrows indicate the corresponding values of F_ST_ for the candidate variant. The *y*-axis represents the frequency of SNPs with a given F_ST_ estimate. Significant F_ST_, which is defined as higher than 95^th^ percentile of F_ST_ of all common SNPs of HapMap populations, is indicated by an asterisk.

Analysis of F_ST_ statistics indicated that the frequency of the derived *CYB5R4* rs1325471 and *GAD2* rs2236418 alleles in CEU, and the frequency of the derived *PPARG* rs2920502 allele in ASN are significantly different from those of YRI (Fig. 1; Table 1, *p*<.05). Consistently, analysis of LD showed that the genomic regions surrounding *CDKAL1* rs9368197, *GAD2* rs2236418, and *PPARG* rs2920502 are characterized by long LD blocks in CEU and ASN (Fig. S2, a-c in File S1). On the other hand, there is no appreciable difference in LD block characteristics surrounding *CYB5R4* rs1325471 among populations (Fig. S2d in File S1).

**Figure 2:**
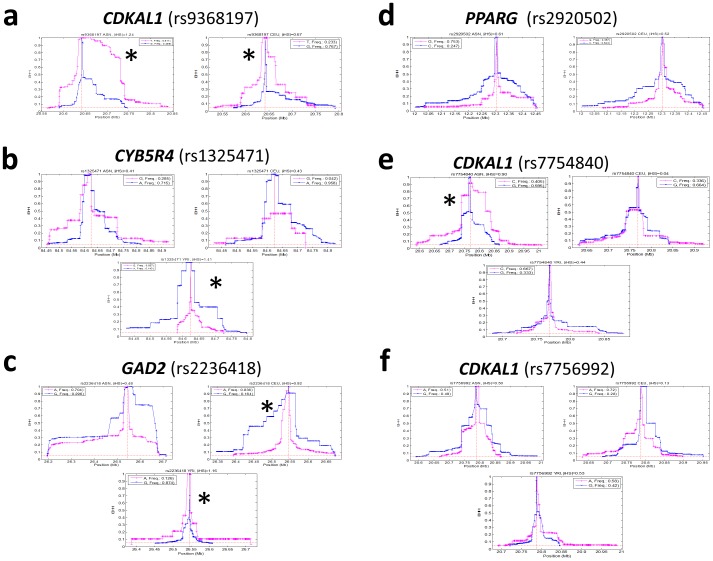
Haplotype diversity surrounding *CDKAL1, CYB5R4*, and *GAD2* variants is allele-dependent in select populations. Plots of iHS over the distance between selected variants and neighboring SNPs at increasing distances in YRI, CEU, and ASN populations (rs9368197 [**a**], rs1325471 [**b**], rs2236418 [**c**], rs292502 [**d**], rs7754840 [**e**], and rs7756992 [**f**]). A significant difference in EHH decay between the derived allele and the ancestral allele is indicated by an asterisk. The candidate variant is positioned at the center of the plots.

Measurements of the iHS statistic using coalescent simulations in HapMap populations with a >10% derived allele frequency showed that the derived *CDKAL1* rs9368197 allele exhibits significant EHH as compared to the ancestral allele in CEU and ASN [4], [18](Fig. 2a). The derived *CDKAL1* rs9368197 allele-associated haplotypes spanned approximately 150 kb, and the frequency of two 90-kb-long derived haplotypes increased from negligible in YRI to more than 53% in ASN (Fig. S3 in File S1, upper panel). Likewise, the ancestral *CYB5R4* rs1325471 allele exhibited EHH in the YRI (Fig. 2b) whereas the ancestral and derived alleles at rs2236418 of *GAD2* exhibited EHH in CEU and YRI, respectively (Fig. 2c). By contrast, rs2920502 of *PPARG* did not exhibit appreciable EHH (Fig. 2d).

Because two intronic *CDKAL1* variants (rs7754840 and rs7756992) that are positioned close to rs9368197 have been associated with type 2 diabetes and GDM in GWA studies [25], [26], [27], [28], we also analyzed the genetic patterns surrounding these two variants (Fig. 2, e-f) [8], [9], [25], [26]. Although the allele frequencies of these variants were similar among HapMap populations (Table 1), the derived allele of rs7754840, but not rs7756992, exhibited a significant EHH in ASN (Fig. 2e).

Taken together, these data indicated that directional selection resulted in the spread of the derived rs9368197 and rs7754840 alleles of *CDKAL1*, the ancestral rs2236418 allele of *GAD2*, and the derived rs2920502 allele of *PPARG* in CEU and/or ASN populations. On the other hand, the ancestral *CYB5R4* rs1325471 allele and the derived *GAD2* rs2236418 allele were selected in YRI populations.

### Common *CDKAL1* variants rs9368197 and rs7754840 impart variations in glucose-induced insulin and GIP response, respectively, in pregnant women

Because pregnancy represents a critical life stage that subjects individuals to excessive metabolic load, and its success has a major impact on reproductive fitness, we chose to analyze the relationships among candidate variants and serum levels of GIP, insulin, and C-peptide in pregnant women with the hope that it would provide a sensitive model to uncover potential genotype–phenotype relationships.

**Figure 3:**
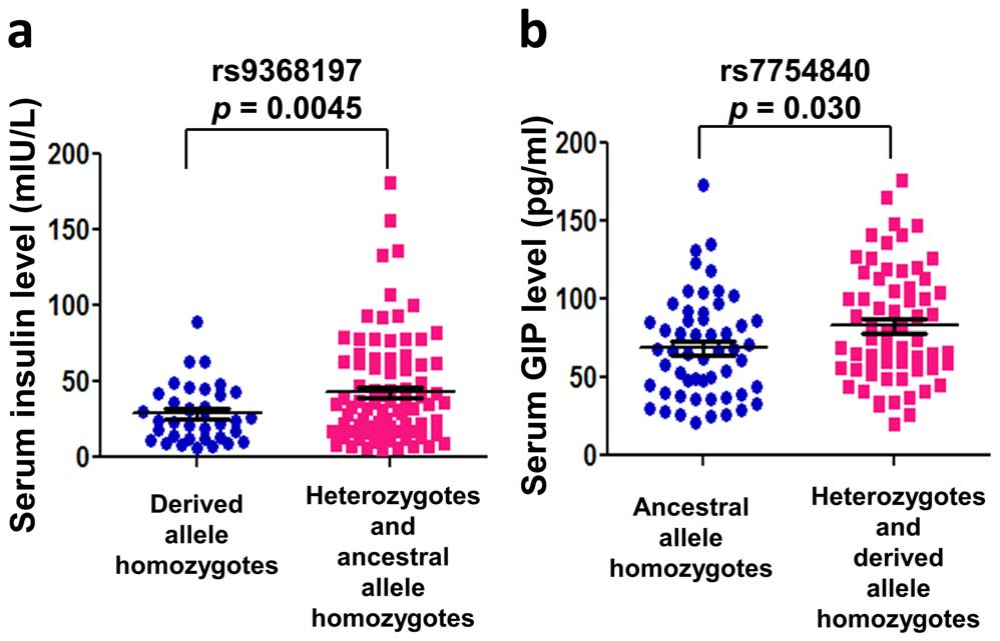
Genotypes of rs9368197 and rs7754840 in *CDKAL1* impart a difference in glucose-induced insulin and GIP response, respectively, after glucose challenge tests. **a**) Measurements of serum insulin levels in patients during the 23^rd^ to the 29^th^ weeks of pregnancy at 1 hr after the 50-gram glucose challenge test. Levels of insulin in patients who were homozygous for the derived T allele of rs9368197 (28.8±3.1 mU/L, *N* = 36) were significantly lower than those of patients carrying the ancestral G allele (i.e., heterozygotes and ancestral allele homozygotes, 42.8±3.7 mU/L, *N* = 89). **b**) Measurements of serum GIP levels in patients during the 23^rd^ to the 29^th^ weeks of pregnancy at 1 hr after the 50-gram glucose challenge test. Circulating levels of GIP in patients who were homozygous for the ancestral G allele of rs7754840 (68.9±4.5 pg/ml, *N*  = 54) were significantly lower than were those of patients carrying a derived C allele (i.e., heterozygotes and derived allele homozygotes, 82.8±4.5 pg/ml, *N* = 65). Serum hormone levels are presented as mean ± SEM.

The frequency of candidate SNPs in the study population was similar to that in ASN (Table 2), and was in the Hardy–Weinberg equilibrium. Measurements of serum hormone levels showed that levels of insulin, but not GIP or C-peptide, in patients who are homozygous for the derived T allele of rs9368197 (i.e., *CDKAL1^−1305T/T^*, *N* = 36) are significantly lower than those carrying an ancestral rs9368197 allele (i.e., *CDKAL1^−1305G/G^* and *CDKAL1^−1305G/T^*, *N* = 89)(Fig. 3a; Table 2).

**Table 2:** Association between selected SNPs and hormonal responses after glucose challenge tests in pregnant women.

Gene (Candidate SNPs)	Frequency of alleles (anc/der)	Insulin (mU/L)	GIP (pg/ml)	C-peptide (ng/ml)
		Mean ± SD [N] (*p* value)^a^		
***CDKAL1*** (rs9368197)	G 0.452/T 0.548	GG 38.5±30.4 [24] (0.953)	GG 82.3±38.7 [25] (0.386)	GG 2.04±1.07 [25] (0.890)
		GT 44.5±38.8 [65]	GT 75.2±34.6 [59]	GT 2.19±1.18 [66]
		**TT 28.8±18.8 [36]** ** (0.005)** b	TT 74.4±34.0 [36] (0.674)	TT 1.87±0.71 [37] (0.111)
***CYB5R4*** (rs1325471)	A 0.300/G 0.700	AA 35.8±22.2 [13] (0.570)	AA 81.9±27.8 [14] (0.500)	AA 2.03±1.05 [14] (0.857)
		AG 40.0±33.3 [49]	AG 75.1±38.8 [46]	AG 2.11±1.06 [50]
		GG 39.7±34.5 [63] (0.917)	GG 77.1±34.8 [59] (0.952)	GG 2.07±1.07 [64] (0.890)
***GAD2*** (rs2236418)	G 0.290/A 0.710	GG 48.3±49.1 [10] (0.523)	GG 72.2±42.1 [9] (0.637)	**GG 1.45±0.79 [10]** ** (0.027)** c
		GA 39.4±29.1 [53]	GA 80.2±36.0 [51]	GA 2.12±1.13 [52]
		AA 36.6±30.8 [63] (0.456)	AA 78.5±41.7 [62] (0.940)	AA 2.12±1.00 [66] (0.571)
***PPARG*** (rs2920502)	C 0.222/G 0.778	CC 33.9±24.4 [6] (0.601)	CC 85.2±46.4 [6] (0.666)	CC 1.70±1.50 [6] (0.785)
		CG 43.8±35.2 [44]	CG 83.1±38.9 [44]	CG 2.26±1.08 [45]
		GG 37.2±31.7 [76] (0.378)	GG 72.3±30.8 [71] (0.103)	GG 2.00±1.00 [77] (0.319)
***CDKAL1*** (rs7754840)	G 0.669/C 0.331	GG 36.1±29.7 [57] (0.394)	**GG 68.9±32.8 [54]** ** (0.030)** c	GG 2.02±0.95 [59] (0.606)
		GC 42.0±36.4 [52]	GC 78.0±34.4 [50]	GC 2.19±1.21 [53]
		CC 37.6±23.5 [15] (0.851)	**CC 98.9.±37.4 [15]** ** (0.026)** b	CC 1.84±0.83 [15] (0.485)

a *p* values of comparisons between a homozygous genotype, and those with a heterozygous or an opposite homozygous genotype (Student's *t*-test).

b Significantly different from the averages of those with a heterozygous or a homozygous ancestral genotype. (*p*<0.05).

c Significantly different from the averages of those with a heterozygous or a homozygous derived genotype (*p*<0.05).

On the other hand, circulating GIP level in patients who are homozygous for the ancestral G allele of rs7754840 (i.e., *CDKAL1^+12656G/G^*, *N* = 54) was significantly lower than that of those carrying a derived C allele (i.e., *CDKAL1^+126562C/C^* and *CDKAL1^+126562G/C^*, *N* = 65)(Fig. 3b; Table 2). Consistently, circulating GIP level in patients who are homozygous for the derived rs7754840 C allele (i.e., *CDKAL1^+12656C/C^*, *N* = 15) was significantly higher than that of those carrying an ancestral G allele (i.e., *CDKAL1^+126562G/G^* and *CDKAL1^+126562G/C^*, *N* = 104)(Table 2). In addition, linear regression analysis showed that the derived *CDKAL1* rs7754840 allele is associated with an enhanced glucose-induced GIP response with an R^2^ = 0.046 (*p* = 0.018).

Furthermore, patients who are homozygous for the ancestral G allele of *GAD2* rs2236418 (*GAD2^−243G/G^*, *N* = 10) had a significantly lower circulating level of C-peptide compared with those carrying a derived A allele (*GAD2^−243G/A^* and *GAD2^−243A/A^*, *N* = 118)(Table 2). By contrast, the *CBY5R4* and *PPARG* variants did not have any significant relationship with serum insulin, GIP, or C-peptide levels.

### The type 2 diabetes/GDM risk variant of *CDKAL1* rs7754840 emerged ∼6,900 years ago in East Asians

**Figure 4:**
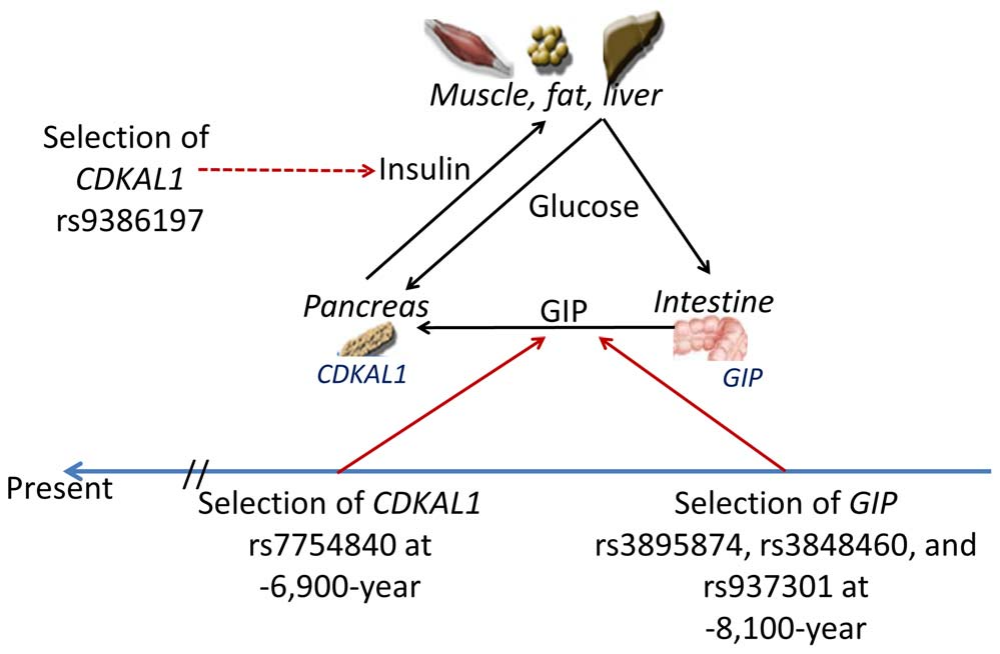
*CDKAL1* and *GIP* variants were selected in recent human history. Schematic representation of the selection of multiple *CDKAL1* and *GIP* variants in ASN population [5], [6]. The selection of *CDKAL1* and *GIP* variants appeared to occur independently within a 1,200-year time window in the last ten thousand years. The effects of these selections on enteroinsular hormonal responses are indicated by red arrows. The actions of hormones are indicated by black arrows.

To better understand putative events that led to the selection of *CDKAL1* variants, we estimated the age of selection based on coalescent simulations [20], [21], [29], [30]. Scanning of the LD map and haplotype structures showed that informative SNPs surrounding rs9368197 are too sparse for reliable age determination. On the other hand, rs7754840 is located in a 151-kb-long LD block, bound between rs6927481 and rs7741604 in the ASN (Fig. S1 in File S1, upper left panel). Using the recombination map of the HapMap project [14], [22], we interpolated the values of accumulative recombination at the positions of the two boundary SNPs of the LD block and took the difference between the two values (0.0946 cM; i.e., 0.0946% chance of crossing over per generation) as the recombination fraction for the region. This was translated into a recombination rate *r* = 9.46×10^−4^. Using the 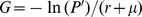 equation, we obtained a generation estimate *G* = 276. An estimated generation time of 25 years indicated that the age of selection for rs7754840 is ∼6,900 years (Fig. 4).

## Discussion

Based on an analysis of the evolutionary history of candidate genes, we showed that common 5′ variants in *CDKAL1*, *CYB5R4, GAD2*, and *PPARG* as well as an intronic type 2 diabetes-associated *CDKAL1* variant exhibit non-neutral evolutionary patterns. In addition, we found that *CDKAL1* and *GAD2* variants are associated with glucose-induced insulin, GIP, or C-peptide responses in pregnant women. Together with our earlier studies of adaptive *GIP* variants [5], [6], these data provided strong evidence that the GIP-insulin-glucose axis is a hotspot for energy-balance regulation-associated selection (Fig. 4) [31].

A wide spectrum of physiological differences among humans are believed to be the result of positive selection of pre-existing variants and recent mutations that accumulated in response to environmental or cultural changes [4], [32]. Recent advances have revealed adaptive SNPs that are associated with immune responses, changes in body forms (e.g., that affect the pigmentation of skin, hair, or eyes), and adaptations to extreme climates (e.g., the selection of the *EPAS1* gene in Tibetans) after humans spread to various parts of the world in the last 50–60 thousand years [1], [2], [3], [4]. However, the identification of adaptive variants in energy-balance regulation appeared to lag behind that of other traits [33], [34]. Earlier studies have shown that variants in *TCF7L2* were positively selected, and were associated with BMI and levels of ghrelin and leptin; however, no general enrichment of adaptive variants in type 2 diabetes- and obesity-associated loci have been found [35]. On the other hand, a study of SNPs in 40 major human diseases indicated that a repertoire of type 2 diabetes risk alleles have a tendency for directional selection in Eurasians [36]. It was speculated that this phenomenon could be associated with culture changes; however, the importance of this ensemble of “selection signal” in humans remains to be investigated. Consistent with these earlier studies, we found only a few SNPs that exhibit signals of selection. The paucity of adaptive SNPs in energy-balance regulation-associated genes could be due to heterogeneity in intensity, form, or time of environmental selection. In addition, recent culture changes that have subjected humans to varying degrees of population growth, and cycles of feast and famine could have made such variants retain only a minimal “signature of selection” [37].

The finding that insulin levels are *CDKAL1* variant-dependent is not new. Intronic *CDKAL1* variants rs7754840, rs7756992, and rs10946398 have been associated with variations in insulin release, pancreatic cell functions, hemoglobin A1C level, and/or response to pancreatic KATP channel agonists [8], [38], [39] as well as type 2 diabetes, GDM, ulcerative colitis, Crohn's disease, obesity, and/or birth weight [9], [25], [26], [28]. CDKAL1 has been shown to function as a tRNA modification enzyme, and its activity is associated with ATP generation and first-phase insulin secretion [40], [41], [42]. The carriage of *CDKAL1* risk variants may lead to lower insulin release and impaired conversion of proinsulin to insulin in pancreatic beta cells [43], [44], [45]. Although the effect of derived rs9368197 allele on glucose-induced insulin response is similar to that reported for the derived rs7754840 allele, the finding that the derived rs7754840 allele (i.e., C) is associated with an elevated GIP response is unique because the same allele has been found to be associated with type 2 diabetes and reduced insulin secretion in earlier studies [43], [44], [45], [46]. These results suggest that the selection of derived rs7754840 allele has opposite effects on insulin secretion and glucose-induced GIP response. Because GIP's normal function is to stimulate insulin secretion, the adverse effect of derived rs7754840 allele on insulin metabolism may be counteracted by that on glucose-induced GIP response. Therefore, the reported association between *CDKAL1* variants and insulin metabolism could be partially affected by the rs7754840-associated GIP responses. In addition, the selection of the derived rs7754840 allele in East Asians may explain part of the observed differences in association strength between *CDKAL1* and type 2 diabetes among human populations [46]. In corroboration with this idea, recent studies have reported that circulating GIP levels are highly familial compared to circulating levels of glucose, insulin, or C-peptide [47]. Future study of the effect of CDKAL1 on GIP synthesis/secretion is needed to reveal whether CDKAL1 affects insulin secretion and GIP metabolism via a similar mechanism.

In a recent investigation, we documented the strong selection of a cluster of *GIP* variants in East Asians ∼8,100 (11,800 to 2,000) years ago [5], [6]. It is surprising to find that the selection of rs7754840 in *CDKAL1* occurred in the same population at approximately the same time period (i.e., ∼6,900 years ago). Because the selection of *CDKAL1* and *GIP* variants overlapped, and because GIP metabolism is highly regulated during pregnancy [48], we speculate that the regulation of enteroinsular axis in Eurasians was subject to strong environmental selection in recent human history (Fig. 4). Because no significant selection of SNPs was observed in other key enteroinsular regulatory genes—including insulin, insulin receptor, glucagon-like peptide-1 (GLP-1), GLP-1 receptor, glucagon, glucagon receptor, and GIP receptor—*CDKAL1* and *GIP* appear to be unique targets for energy-balance regulation-associated selections.

Although it is impossible to discern which environmental forces are responsible for the selection of *CDKAL1* and *GIP* variants, the selection of these variants during a period that heralded the agriculture revolution and animal domestication (i.e., 10,000 to 4,000 years ago following the Neolithic period) suggests that the selected *CDKAL1* and *GIP* variants may have facilitated the survival of their carriers in the face of changing subsistence culture. As culture again shifted in modern society, some of these prior advantageous adaptations (e.g., rs7754840) now may manifest as risk factors for type 2 diabetes and GDM [25], [27], [28], [44], [45]. In support of this view, recent studies have shown that minimal changes in subsistence patterns could have profound effects on the survival of individuals [49]. For example, it has been shown that feeding mice human-relevant concentrations of added sugar (i.e., 25% kcal from a mixture of fructose and glucose) is sufficient to increase mortality and reduce fertility even though such treatments have minimal effects on serum levels of cholesterol, fasting insulin, fasting glucose, or fasting triglycerides. Although our hypothesis cannot be falsified at the present time, future studies that consider Neanderthal and Denisovan genomes may provide further insight [50].

Studies of hormonal profiles have also uncovered an association between *GAD2* rs2236418 and glucose-induced C-peptide response. Earlier studies have shown that polymorphisms of *GAD2*, which encodes an enzyme that catalyzes the production of gamma-aminobutyric acid (GABA), are associated with obesity, BMI, and postabsorptive resting energy expenditure in selected populations [10], [51], [52], [53], [54]. GABA originating within the islets can evoke tonic currents, and decrease both insulin and glucagon secretion [55], [56]. In addition, the GABA signaling system has been shown to be compromised in islets of type 2 diabetes patients [57]. Therefore, the adaptive *GAD2* variant could indirectly contribute to variations in C-peptide, and perhaps insulin, metabolism by affecting GABA synthesis. Future study of the rs2236418-C-peptide relationship in other populations is needed to have a better understanding of the regulation of enteroinsular axis via neuronal signaling in humans.

Whereas *CYB5R4* and *PPARG* variants were not associated with the limited number of hormones that were analyzed, the “signals of selection” surrounding these variants warrant future investigations under different conditions. Among them, rs2920502 and linked SNPs like rs2920500 could be particularly interesting because *PPARG* has been associated with the development of type 2 diabetes, atherosclerosis, and cancer [7], [58], and because PPAR-γ agonists (e.g., thiazolidinedione) have been used to treat type 2 diabetes [58]. Likewise, future studies in patients of African or European origin may further reveal the role of selected *CYB5R4* and *GAD2* variants in the regulation of hormonal metabolism because these variants were selected in CEU and/or YRI.

In conclusion, the present study showed that adaptive *CDKAL1* variants could spread in Eurasians by contributing to alternative insulin or GIP responses. Therefore, environmental or culture changes in recent human history could have shaped the regulation of our enteroinsular axis to a greater extent than what has previously been assumed.

## Supporting Information

File S1 **Figure S1. Plots of the haplotype structure of SNPs in a 151-kb LD block surrounding rs7754840 in the HapMap II populations.** The 70 SNPs between rs6927481 and rs7741604 (151-kb in length) were linked, and displayed a low haplotype diversity in the ASN population (left upper panel). By contrast, the same region in the YRI chromosomes (right panel) exhibited a high complexity compared to the CEU and ASN populations. The position of rs7754840 is indicated by a vertical rectangular box. **Figure S2. Variants in** ***CDKAL1, GAD2*** **, and** ***PPARG*** **are highly linked in select human populations.** Plots of the degree of LD between each pair of genotyped SNPs in a 200-kb region surrounding the *CDKAL1* (a), *GAD2* (b), *PPARG* (c), and *CYB5R4* (d) loci in YRI, CEU, and ASN populations. The color scheme was based on r^2^ values. Red areas represent regions with a high degree of LD and a high likelihood of odds (LOD) (D' = 1, LOD scores >2). Blue areas represent regions with low LOD (D' = 1, LOD <2). **Figure S3. Haplotype structures of SNPs neighboring rs9368197 in the HapMap II populations.** Plots of the haplotypes in a 200-kb genomic region (20,541-20,741 kb on chromosome 6) surrounding rs9368197 in *CDKAL1* showed that ASN chromosomes (upper panel) are characterized by a low-complexity structure whereas YRI chromosomes exhibit extensive recombinations (lower panel). The CEU chromosomes exhibited an intermediate pattern of complexity (middle panel). A 90-kb region with extensive haplotype homozygosity in ASN chromosomes is indicated by a red rectangular box in each of the three haplotype structure plots. The position of rs9368197 in each plot is indicated by a blue arrow.
(PDF)
