# Supplementary material for: Adaptive Human CDKAL1 Variants Underlie Hormonal Response Variations at the Enteroinsular Axis
Source: PLoS One. 2014 Sep 15;9(9):e105410. doi: 10.1371/journal.pone.0105410 (PMC4164438; doi:10.1371/journal.pone.0105410)
Supplement: File S1 — Figure S1. Plots of the haplotype structure of SNPs in a 151-kb LD block surrounding rs7754840 in the HapMap II populations. The 70 SNPs between rs6927481 and rs7741604 (151-kb in length) were linked, and displayed a low haplotype diversity in the ASN population (left upper panel). By contrast, the same region in the YRI chromosomes (right panel) exhibited a high complexity compared to the CEU and ASN populations. The position of rs7754840 is indicated by a vertical rectangular box. Figure S2. Variants in CDKAL1, GAD2 , and PPARG are highly linked in select human populations. Plots of the degree of LD between each pair of genotyped SNPs in a 200-kb region surrounding the CDKAL1 (a), GAD2 (b), PPARG (c), and CYB5R4 (d) loci in YRI, CEU, and ASN populations. The color scheme was based on r2 values. Red areas represent regions with a high degree of LD and a high likelihood of odds (LOD) (D' = 1, LOD scores >2). Blue areas represent regions with low LOD (D' = 1, LOD <2). Figure S3. Haplotype structures of SNPs neighboring rs9368197 in the HapMap II populations. Plots of the haplotypes in a 200-kb genomic region (20,541-20,741 kb on chromosome 6) surrounding rs9368197 in CDKAL1 showed that ASN chromosomes (upper panel) are characterized by a low-complexity structure whereas YRI chromosomes exhibit extensive recombinations (lower panel). The CEU chromosomes exhibited an intermediate pattern of complexity (middle panel). A 90-kb region with extensive haplotype homozygosity in ASN chromosomes is indicated by a red rectangular box in each of the three haplotype structure plots. The position of rs9368197 in each plot is indicated by a blue arrow. (PDF) [file pone.0105410.s001.pdf]

ASN

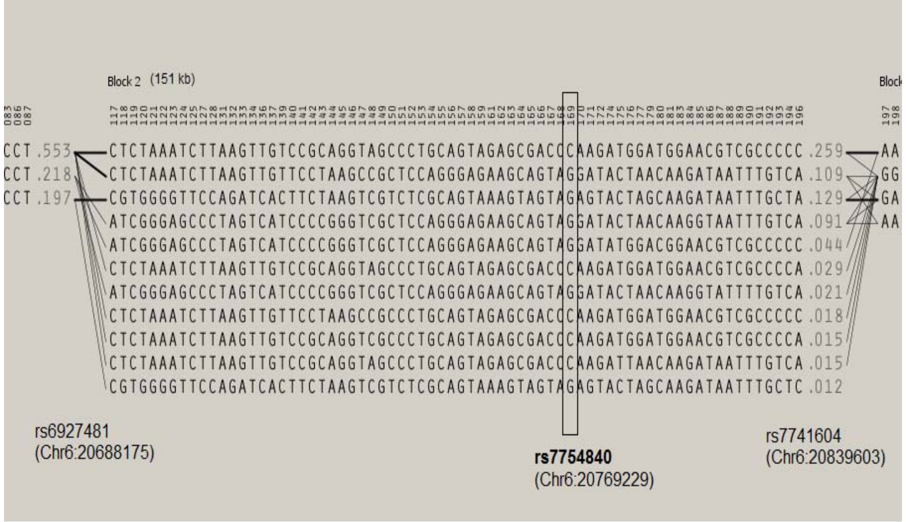

CEU

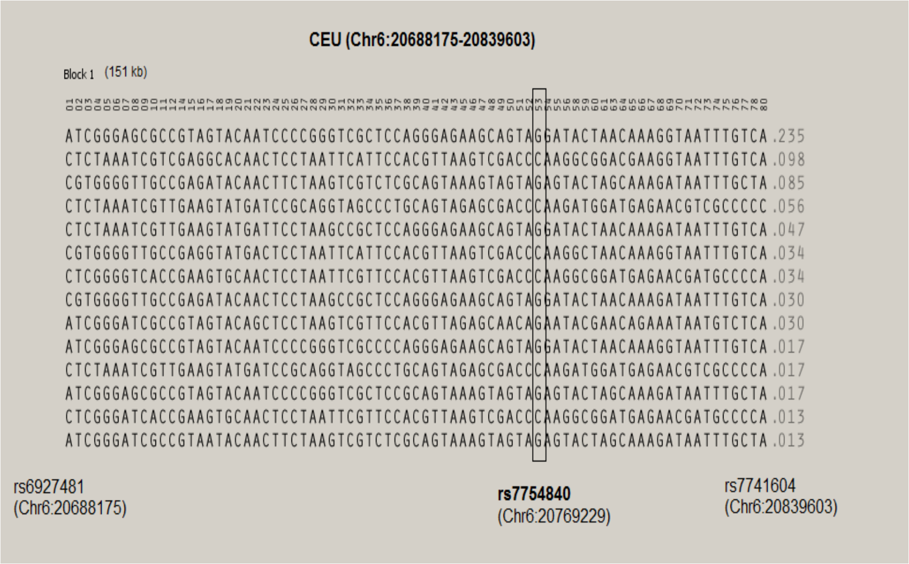

YRI

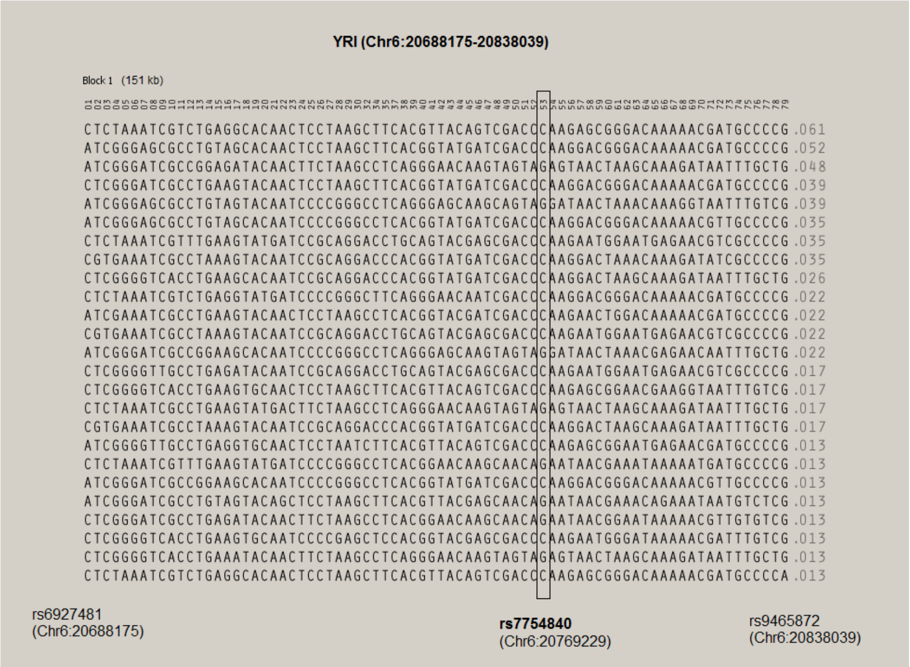

Fig. S1

CDKAL1 rs9368197

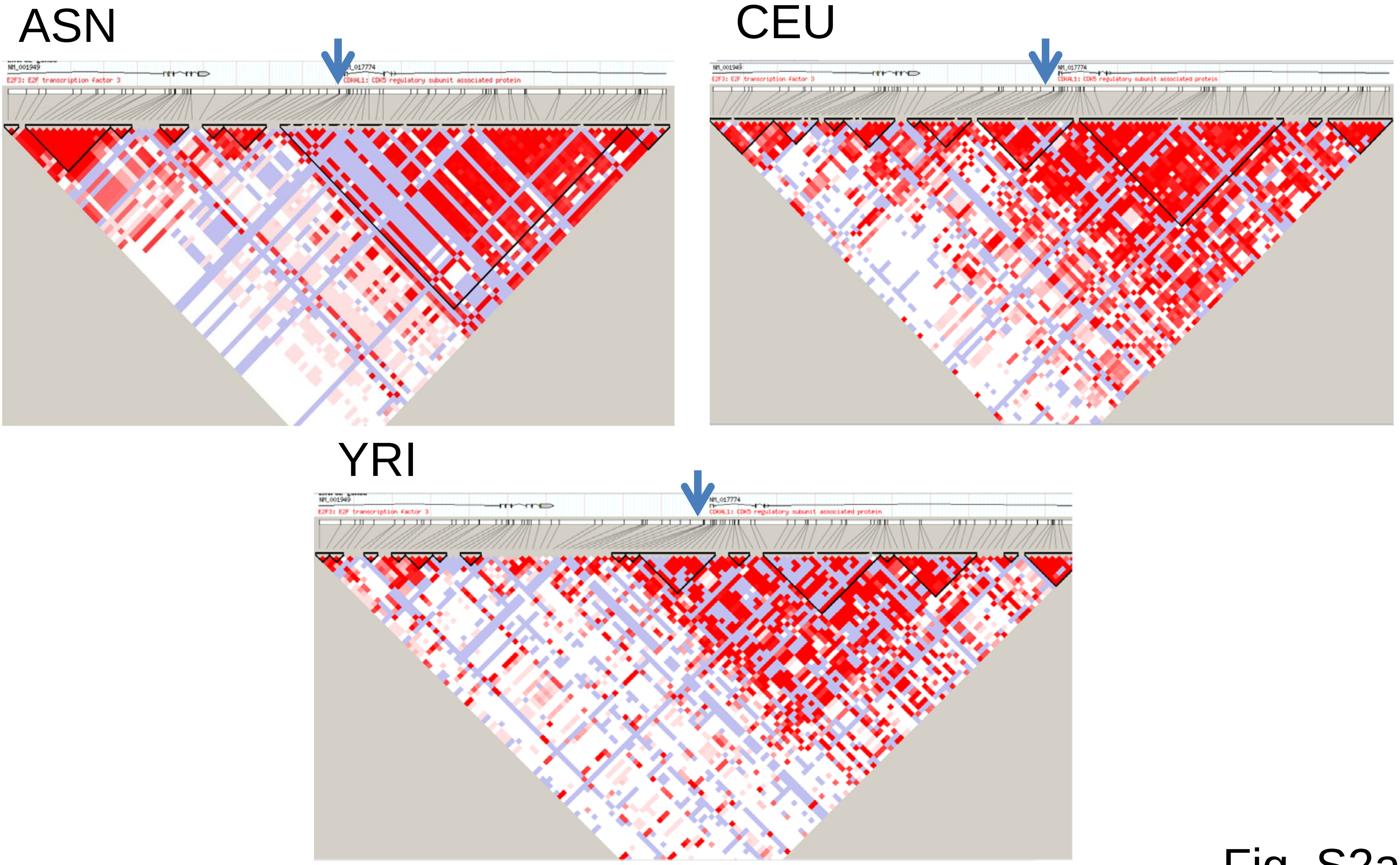

Fig. S2a

GAD2 rs2236418

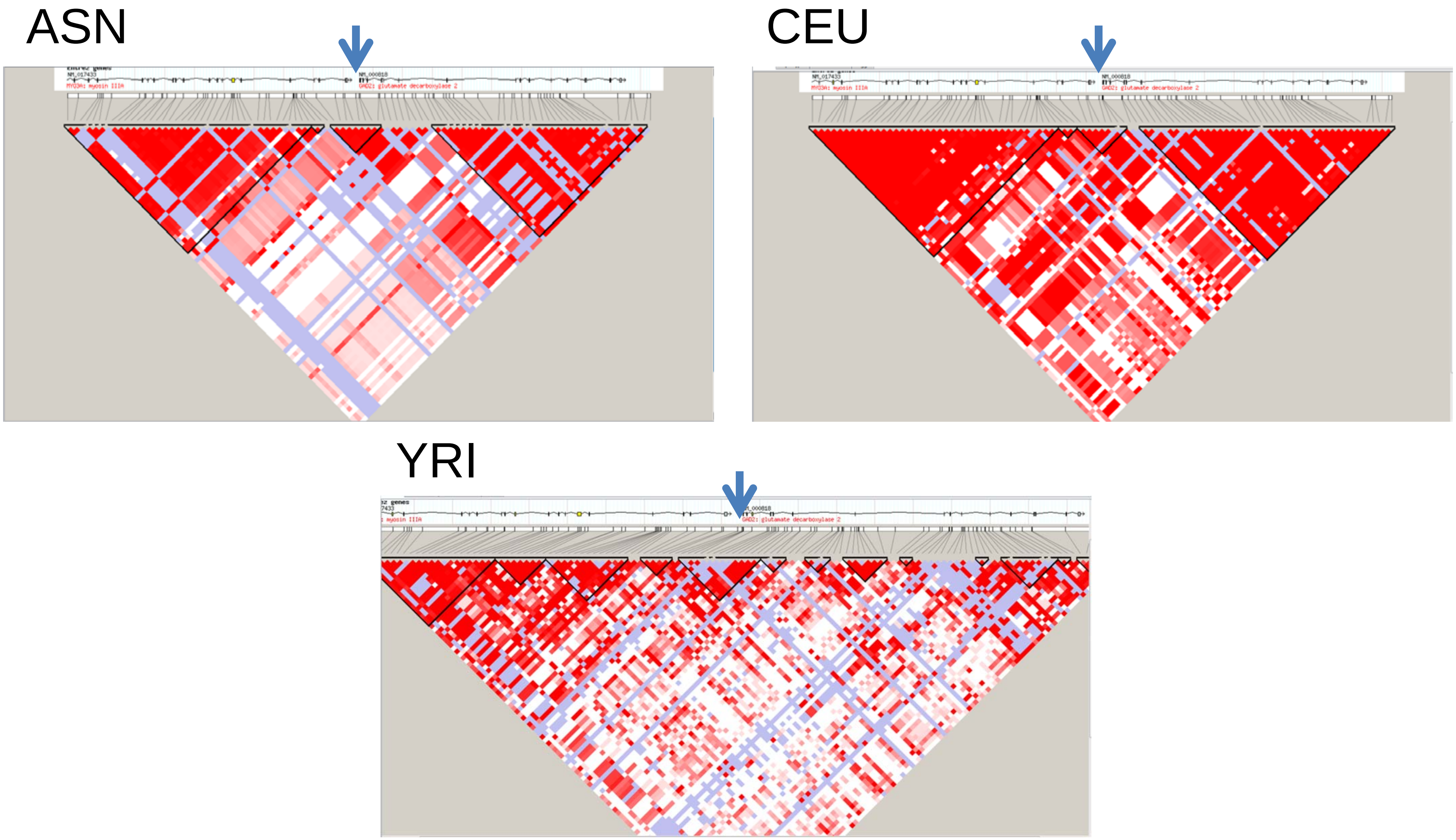

Fig. S2b

PPARG rs2920502

ASN

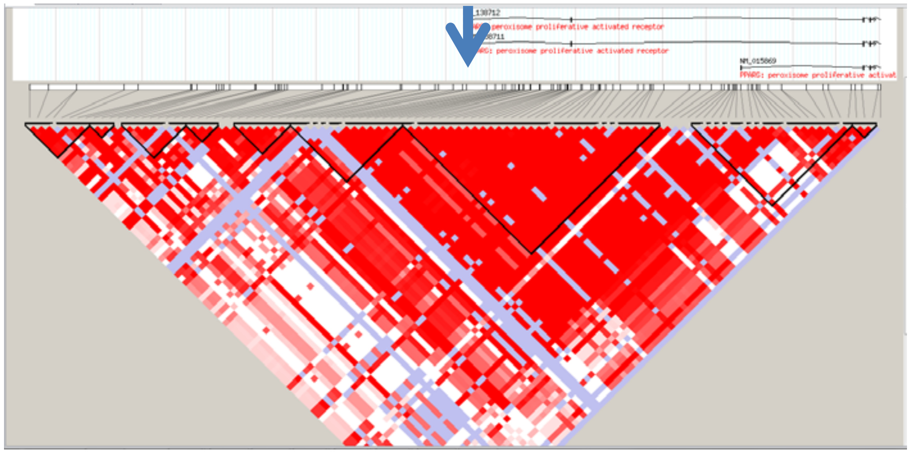

CEU

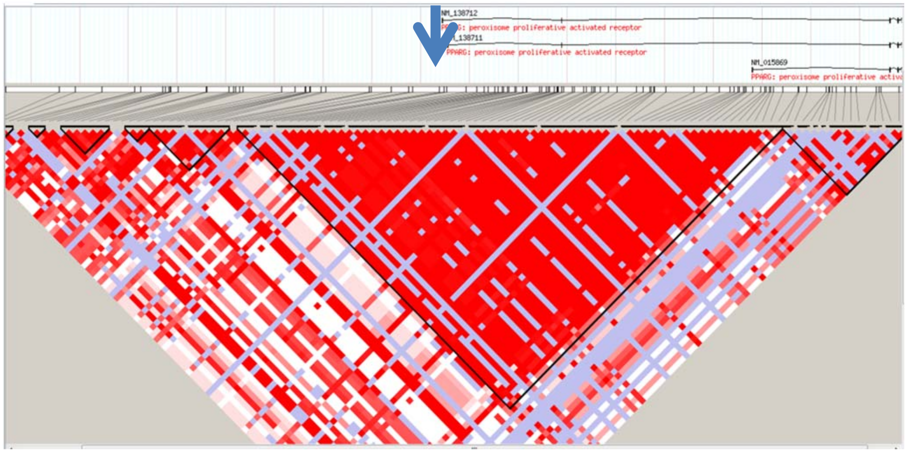

YRI

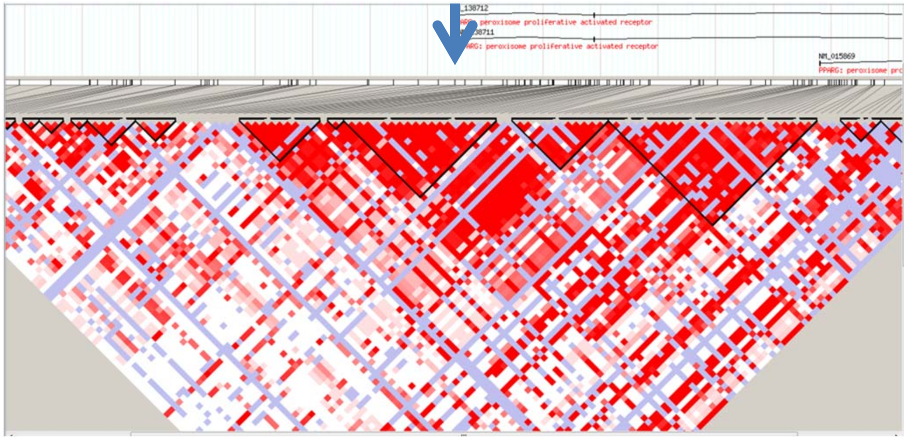

Fig. S2c

CYB5R4 rs1325471

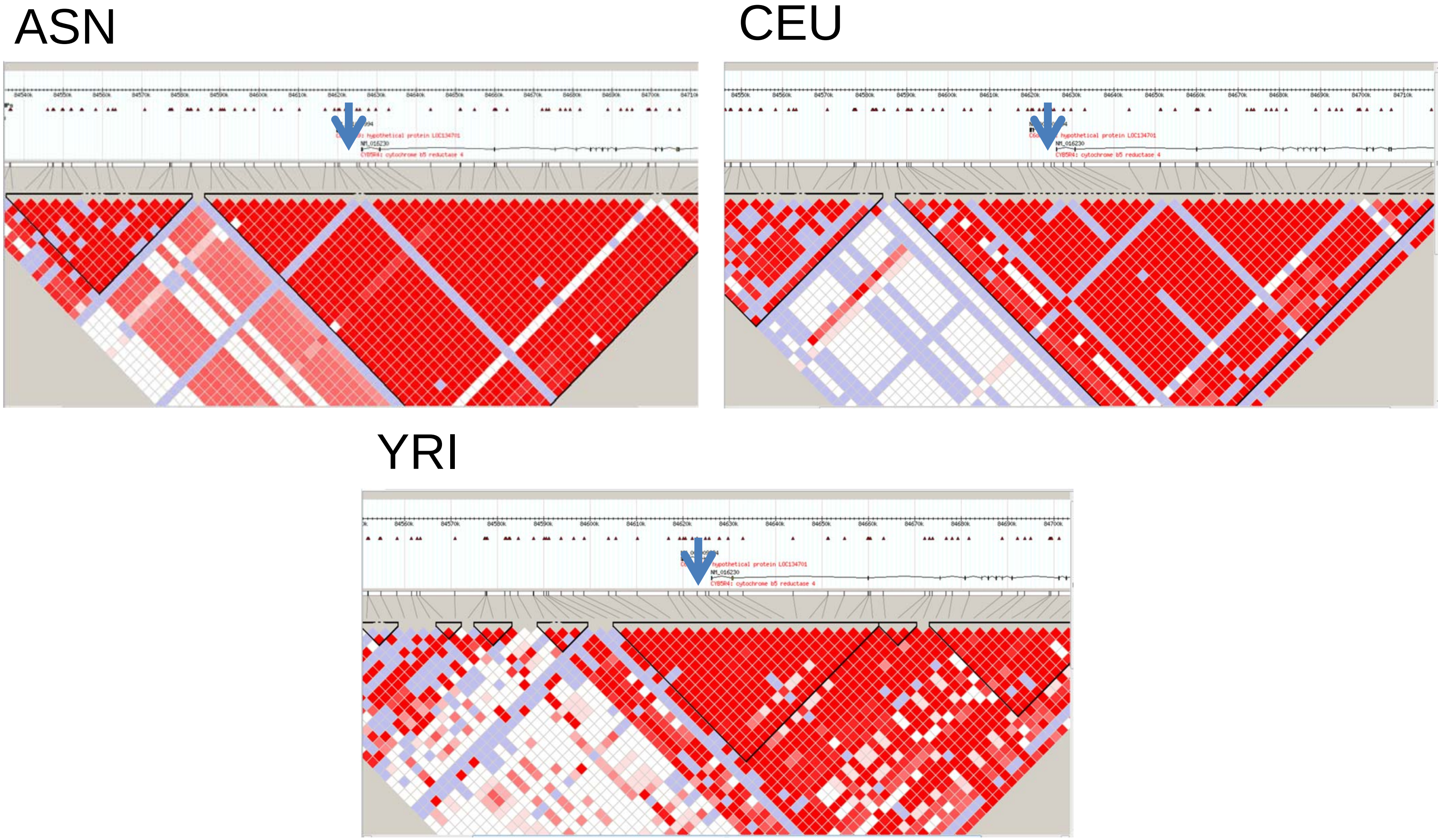

Fig. S2d

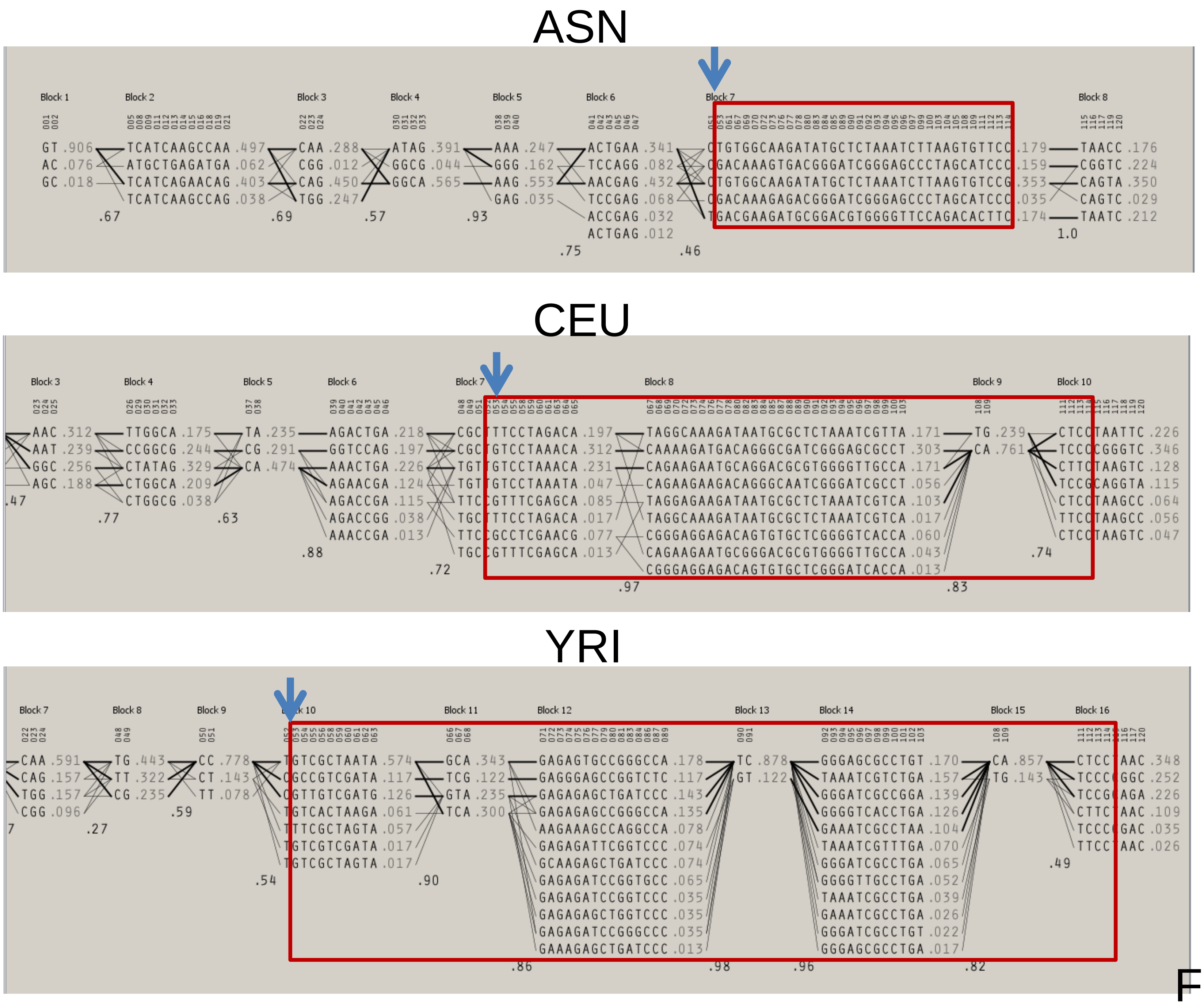
ASN
Block 1
Block 2
Block 3
Block 4
Block 5
Block 6
Block 7
Block 8
CEU
Block 3
Block 4
Block 5
Block 6
Block 7
Block 8
Block 9
Block 10
YRI
Block 7
Block 8
Block 9
Block 11
Block 12
Block 13
Block 14
Block 15
Block 16

Fig. S3
